# Acute Effects of Caffeine on Overall Performance in Basketball Players—A Systematic Review

**DOI:** 10.3390/nu14091930

**Published:** 2022-05-05

**Authors:** Anja Lazić, Miodrag Kocić, Nebojša Trajković, Cristian Popa, Leonardo Alexandre Peyré-Tartaruga, Johnny Padulo

**Affiliations:** 1 Faculty of Sport and Physical Education, University of Niš, 18000 Niš, Serbia; anja.lazic96@hotmail.com (A.L.); miodrag.kocic73@gmail.com (M.K.); nele_trajce@yahoo.com (N.T.); 2 Faculty of Physical Education and Sport, Ovidius University of Constanta, 900470 Constanta, Romania; crispopa2002@yahoo.com; 3 Exercise Research Laboratory, Universidade Federal do Rio Grande do Sul, Porto Alegre 90690-200, RS, Brazil; leonardo.tartaruga@ufrgs.br; 4 Department of Biomedical Sciences for Health, Università degli Studi di Milano, 20133 Milan, Italy

**Keywords:** supplementation, nutrition, team sport, explosive power, agility, speed

## Abstract

Caffeine supplementation has become increasingly popular among athletes. The benefits of caffeine include delaying the negative effects of fatigue, maintaining a high level of physical and mental performance, and improving certain abilities necessary for sport success. Given the complex nature of basketball, caffeine could be a legal, ergogenic stimulant substance, which will positively affect overall basketball performance. The purpose of this systematic review was to summarize evidence for the effect of acute caffeine ingestion on variables related to the basketball performance. Web of Science, PubMed, Scopus and ProQuest, MEDLINE, and ERIC databases were searched up to February 2021. Studies that measured the acute effect of caffeine on basketball performance were included and analyzed. Eight studies published between 2000 and 2021 were included in the analysis. Pre-exercise caffeine intake increased vertical jump height, running time at 10 and 20 m without the ball, overall basketball performance (number of body impacts, number of free throws, rebounds, and assists) during simulated games, and reduced the time required to perform a basketball-specific agility test. Equivocal results between caffeine and placebo groups were found for aerobic capacity, free throw and three-point accuracy, and dribbling speed. Pre-exercise caffeine ingestion did not affect RPE, but insomnia and urinary excretion were increased. The pre-exercise ingestion of 3 and 6 mg/kg caffeine was found to be effective in increasing several physical performance variables in basketball players during sport-specific testing and simulated matches. However, considering the intermittent nature and complexity of basketball, and individual differences between players, future studies are needed.

## 1. Introduction

Basketball players have been affected by very high internal and external loads during training and competition [1]. Performance implies a large volume of high-intensity activities of short duration—speed, strength, agility, and endurance [2]. These activities represent the basis for the performance of jumping and sprinting movements in different directions and at different angles, and they are combined with sports-specific elements [3,4]. As a multifactorial game, basketball includes shooting and passing accuracy as an integral, crucial, and most frequent technical part of the activity [5,6]. During a basketball match, players perform over 50 jumps [7], 48.7% of all basketball activities include combination of jumping and shooting movements, and 28.5% imply repeated sprint ability [8]. Additionally, a distance of 4000 to 5000 m is covered within 40 min [9]. The previously highlighted facts state the complex nature of the metabolic systems of basketball, mixing aerobic and anaerobic demands. Performing repeated high-intensity activities during training sessions and games induces a great pressure on the central nervous system (CNS) and causes fatigue [10]. Unquestionably, fatigue may decrease basketball performance [11].

In recent years, researchers have become increasingly interested in a variety of ergogenic substances that can delay the effect of fatigue and improve athletic performance [12]. Moreover, by removing caffeine from the list of prohibited substances [13] this supplement has become the most popular ergogenic substance used by athletes [14]. More precisely, data obtained from urine samples of athletes from different sports show that three out of four athletes consume caffeine-based supplements before or during competition [14]. Most researchers working in the area of caffeine effects on athletic performance agree that caffeine has an important role in performance improvement during endurance activities [15,16,17], as well as in power-related activities [18,19]. In addition, there is strong evidence that the consumption of certain doses of caffeine may even improve performance during repeated sub-maximal and maximal activities [18,20,21]. The main physiological symptoms of caffeine use are respiratory rate and minute volume increases, overstimulation of the respiratory centers, intensifying pulmonary blood flow, and the sensitivity of central medullary areas to hypercapnia. These alterations are mediated by the antagonistic action of caffeine on adenosine receptors [22]. Given that many high-intensity activities are present in basketball, and that their manifestation leads to the presence of acute fatigue, and at the same time to great stress to the CNS, caffeine supplementation could be an adequate solution for maintenance or improvement overall performance in this sport.

Previous studies [23,24,25,26] have explored the role of caffeine intake in team ball games. Recently, in a systematic review conducted by Chia and colleagues [23], conflicting results have been reported in the most popular ball games. Improvements caused by caffeine intake are shown in jumping and running activities, but authors have failed to report positive effects on specific motor abilities—agility and accuracy. This finding is congruent with a later systematic review with meta-analysis [24], which showed that ingestion of caffeine has small but significant effects on several components of physical performance in team sports.

However, previous research in the field of investigation has been restricted to only two studies in basketball and a small sample size. Principally, with this background, it is very difficult to determine whether caffeine is an ergogenic substance for basketball players. In addition to the unclear literature evaluating caffeine’s effect on basketball performance, to the best of our knowledge, there is a limited number of systematic reviews that focused on the acute effects of caffeine on the overall performance in basketball. This paper provides an overview of the effect of acute caffeine ingestion on variables related to basketball performance.

## 2. Materials and Methods

### 2.1. Search Strategy

The following databases were used to collect adequate literature: Web of Science, PubMed, Scopus and ProQuest, MEDLINE and ERIC up to February 2022. A search of databases was carried out using a combination of the keywords: caffeine, caffeinated, basketball, endurance, performance, strength. The PubMed search is shown below:

(Caffeine) OR (caffeinated energy drinks)) OR (caffeinated energy drink)) AND (basketball)) OR (basketball performance)) AND (explosive power)) OR (strength)) OR (speed)) OR (aerobic capacity)) OR (endurance)) OR (flexibility)) OR (agility)) OR (change of direction speed)) OR (precision)) OR (accuracy)

**Figure 1:**
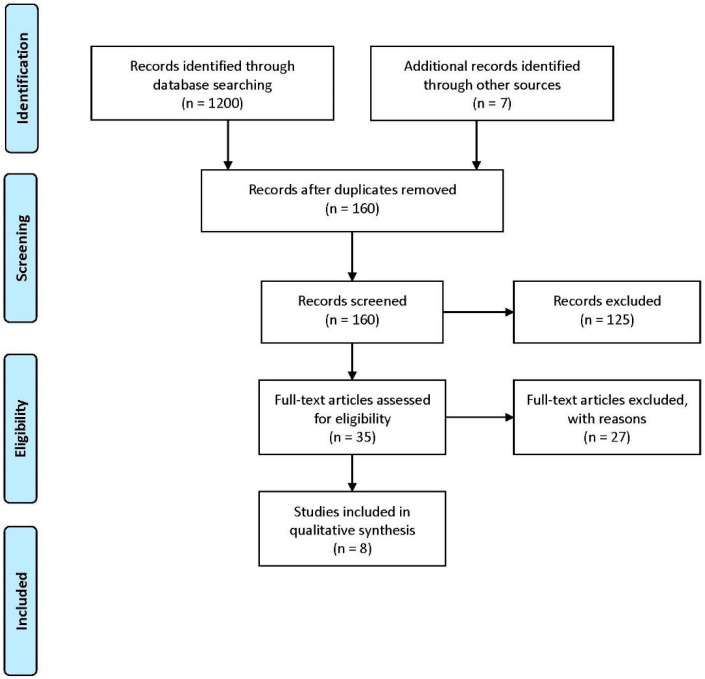
PRISMA preferred reporting items for systematic reviews and meta-analysis.

A systematic review of the available literature was undertaken in accordance with the Preferred Reporting Items for Systematic Reviews and Meta-Analysis (PRISMA) guidelines [27] (Figure 1). All studies that passed the process of previous selection were downloaded to Endnote X9 (Clarivate Analytics, New York, NY, USA) and a subsequent review of the entire text of all studies was performed to remove duplicates. The search strategy was performed by two authors independently (N.T. and A.L.) and study selection was limited to the articles published in English. Disagreements were resolved through discussion of two mentioned authors. The selected studies are presented according to the following parameters in Table 1 and Table 2. Table 1 shows basic information about the study and the participants: (a) reference, (b) participants (gender, number, and age), (c) level of playing, (d) daily dose of caffeine intake through various products, (e) PEDro score rating. Table 2 presents the parameters of the experimental treatment: (a) dose of caffeine, (b) the form in which caffeine is consumed during testing, (c) tested variables, (d) results, (e) results shown by an adequate symbol (↑—improvement, ↓—reduction, ↔—no changes).

### 2.2. Inclusion Criteria

In this systematic review, the following criteria had to be met: (1) studies published in English; (2) studies examining the acute effects of caffeine on basketball performance; (3) studies that were subjected to a randomization process, were controlled, armored and with a cross-over design, and which involved comparing the acute effects of caffeine with the effects of a placebo substance; (4) studies in which the exact dose of caffeine used per kilogram of body mass was precisely explained in the experimental design, as well as studies in which the time period of caffeine/placebo consumption before the experimental program was highlighted; (5) it was necessary that the time interval between tests was at least 24 h; (6) the sample of participants consisted of basketball players of both sexes, regardless of age and competitive rank; (7) studies published in the period from 2000 to 2021; (8) all studies had to be approved by the appropriate Ethics Committee, with the appropriate number and confirmed date of approval.

### 2.3. Exclusion Criteria

The following criteria were applied for studies exclusion from further analysis: (1) studies were not published in English; (2) studies with abstract available only; (3) longitudinal studies with the aim of examining the long-term effects of caffeine supplementation; (4) if the caffeine intervention contained less than 2 mg/kg which showed insufficient ergogenic effects on various forms of exercise; (5) lack of placebo treatment group; (6) studies that did not have basketball players as the participants; (7) All systematic reviews research or systematic reviews with meta-analysis which investigated the acute effects of caffeine in team sports with the ball; (8) the studies which included wheelchair basketball players as the participants.

### 2.4. Quality Assessment of the Experimental Studies

To determine the quality of each experimental program, the Physiotherapy Evidence Database Scale (PEDro) scale was used (Table 1). The PEDro scale is a valid and reliable way to determine the internal validity of randomized measurements [36]. The PEDro scale consists of 11 items that include information on randomization, blinding procedure, statistical analysis data, and presentation of results in the study. Any study that did not meet the criteria, i.e., to which less than six points were assigned during the PEDro scale check, was excluded from further analysis. Two authors (J.P. and M.K.) independently performed methodological quality using the PEDro scale description guidelines, and disagreements were resolved through discussion.

## 3. Results

A total of 1200 studies were extracted by the initial search from the databases search and 7 articles from the list of references. In addition, 1047 studies were excluded due duplicates found, and 160 studies are further screened. We removed 152 studies after full-text review. Finally, in total, eight studies meet the inclusion criteria. The Table 1 and Table 2 highlight general information about the participants and experimental treatment. The eight studies included in this systematic review were published between 2014 and 2021. The total sample consisted of 120 basketball players and the largest number of the participants was n = 21 [32], while the smallest was n = 5 [28]. Of the total number, male participants were included in three studies [28,29,35], while female participants were included in one study only [33]; both male and female participants were included in four studies [30,31,32,34]. The oldest group was 27.9 ± 6.1 years old [30], and the youngest was 14.9 ± 0.8 years old [29]. All participants were low-to-moderate daily caffeine consumers. In seven studies, the caffeine dosage was 3 mg/kg [28,29,30,31,32,33,35] and in one study it was 6 mg/kg [34]. Of the total number of studies, in seven studies, caffeine was taken in the form of a capsule, and in one by consuming an energy drink [29]. The applied dose of caffeine was taken 60 min before testing in all selected studies, and the PEDro scale score ranged from 8 to 10 points and thus, the quality of these studies can be considered to be of moderate to high quality.

## 4. Discussion

The purpose of this systematic review was to summarize the effects of acute caffeine ingestion on variables related to the basketball performance.

### 4.1. Acute Effects of Caffeine on Physical Performance of Basketball Players

The analysis of the results showed that caffeine in doses of 3 mg/kg has a positive effect on improving the height of the vertical jump [29,30,31,33,35] and linear speed at 10 and 20 m without the ball [33]. Subsequently, the dose of 3 mg/kg increased the number of body impacts and overall basketball performance during simulated games [30]. The vertical jump is one of the most important components of the basketball game [37]. The use of 3 mg/kg of caffeine improves the height of the jump in basketball players, which is congruent with previous studies [38,39,40], in which caffeine was the ergogenic substance used for improving vertical jump height. However, it should be mentioned that the increase in the height of the vertical jumping in these studies were obtained by using energy drinks, which contain other stimulant substances in addition to caffeine that may affect performance [41]. The positive effect of caffeine on the vertical jump in basketball was also proven in later studies in which the participants ingested pure caffeine [30,33,35]. The difference is that the effect of the improvement in basketball players was greater than that caused by the energy drinks [29]. Abian-Vicen et al. [29] examined acute caffeine-based drink in young basketball players and found an increase in the vertical jump of 2.1%, which is lower when compared to the increase of 4.6% in countermovement jump, the 3.8% in countermovement jump with arm swing, and the 4.8% in squat jump reported by using pure caffeine [33]. Also, professional players are more susceptible to the ergogenic effects of caffeine when compared to younger players [42], which is one of the reasons for this difference.

Since change of direction speed in basketball is a frequent activity and it is related to all other elements of the game and to the CNS, only two studies [30,33] tried to determine the effects of 3 mg/kg of caffeine on this ability. While Puente et al. [30] did not find statistically significant improvements on the change of direction agility test, Stojanović et al. [33] came to the conclusion that caffeine can reduce the time required to perform this activity. The possible reason for the discrepancy in the obtained results can be explained by the fact that the change of direction agility test cannot be applied to a specific basketball performance [33]. Conversely, the work of Stojanović et al. [33] showed a small improvement in time on the agility test and this result should be considered more relevant because a specific basketball test was used in the testing that reflects the movements present in the game [43]. In favor of this finding, Davis and Green [42] reported that the efficacy of caffeine on anaerobic performance is more effective when the assessment protocol reflects sports demands.

### 4.2. Acute Effects of Caffeine on Specific Basketball Performance

Caffeine as the ergogenic substance can affect the improvement of psychomotor and cognitive functions during fatigue that occurs because of high-intensity activities and thus has an impact on the adequate performance of technical elements [44]. However, research in this area of investigation has failed to explore the acute effects of 3 mg/kg [29,30] and 6 mg/kg [34] on overall accuracy and dribbling speed [32] in basketball players. Even though there was an increased number of free throws, the use of 3 mg/kg caffeine did not result in the improvement of shot accuracy. Specifically, Tan et al. [34] were unable to identify changes in accuracy by using 6 mg/kg of caffeine. More precisely, Tan et al. [34] found that in the state of acute fatigue, considering individual variations, there was no overall improvement in accuracy when performing free throws. Accuracy is a complex ability and depends on the interaction of numerous factors such as the trajectory of the ball after ejection, the phase of the shooting activity, the movements of which each phase consists and postural balance of the player [45], as well as a limited visual field and tactile sensation of the fingertips [46].

In addition to physical performance, caffeine has effects on hormonal, metabolic, and psychological status [47]. Some authors [24] do not recommend the use of caffeine in team sports with the ball, in which technical and tactical elements are a key factor [5,6]. Namely, the use of caffeine leads to insomnia, increased nervousness, and tremor [48,49], which can negatively affect the mentioned parameters. However, although the use of caffeine initiated increased insomnia [30] after the experimental treatment, caffeine also increased the number of rebounds, assists, performance index, and total body impacts [30] by increasing awareness and alertness during testing basketball players. To avoid the negative effects of caffeine such as insomnia, it is better to use it in the morning, which was confirmed in the study by Stojanović et al. [35]. It was concluded that 3 mg/kg of caffeine had effects on improved performance in the morning, compared to afternoon testing. However, it is difficult to apply these results in practice, due to most games and training being performed in the evening.

### 4.3. Dosing, Timing and Individual Responses to Caffeine

The dose of caffeine used in the studies were above 3 mg/kg, suggesting that doses below 2 mg/kg may not be effective for performance in team sport [24,32]. Caffeine, or an adequate placebo, was ingested 60 min before the experimental program, since is it claimed that caffeine needs 30 to 60 min to be absorbed and to reach the plasma concentration peak [50]. The daily use of caffeine in all studies is labeled as low to moderate. However, daily use was measured based on the mean value of the group and it is necessary to emphasize the individual amount of consumption, because caffeine is strongly individually determined and may not have the ergogenic effect in all users [51]. Also, training status, tolerance to caffeine, or genotype variation have been defined as factors that can influence acute caffeine effects [52]. While some limiting factors can be reversed by increasing the dose of caffeine, numerous studies [53,54,55,56] have drawn parallels between an adequate response to caffeine intake and the presence of different genetic variations. However, a study carried out with 21 male and female basketball players found that both AA homozygotes and C-allele carriers similarly increased their physical performance after ingestion of 3 mg/kg of caffeine [30]. Future studies will have to identify mechanisms through which genetic variations correlate with caffeine intake.

### 4.4. Study Limitations

This systematic review presents some limitations related to the variety of performance tests and variables used in the studies included, as well as the lack of studies based on caffeine effects on basketball performance. Secondly, our findings are not generalizable to the entire basketball population due to the small number of the participants. Previously highlighted facts resulted in the impossibility of meta-analysis and more relevant insights into the acute effects of caffeine. However, given that basketball is a complex sport in which physical performance is only one of several factors necessary for succeeding, our findings should not be over-interpreted. Therefore, several findings of this systematic review warrant further discussion, such as the acute effects of caffeine supplementation on sport-specific situations, especially decision-making situations when players are fresh, and during acute fatigue. Moreover, future studies in this area of investigation should test mechanisms involved in the generation of post-activation potentiation with concomitant caffeine ingestion. Therefore, these findings are still incomplete and still not enough to determine whether caffeine improves overall basketball performance.

## 5. Conclusions and Practical Applications

In summary, the pre-exercise ingestion of 3 and 6 mg/kg of caffeine was found to be effective in increasing several physical performance variables in basketball players during sport-specific testing and simulated matches. Caffeine significantly increases vertical jump performance, sprint performance without the ball, planned agility, number of three throws, rebounds, assists, and body impacts during simulated matches. Equivocal results were found for endurance, accuracy, and dribbling speed. This study provides data that will help coaches, nutritionists, and basketball players in resolving doubts about the use of caffeine as a stimulant pre-game or during the matches. Finally, future studies will have to continue to explore the effects of caffeine in situations such as those during the games and, during acute fatigue taking individual differences between players into account.

## Figures and Tables

**Table 1:** Descriptive statistics of the participants and PEDro scale for assessing quality of included studies.

Study	Participants			Level of Playing	Daily Caffeine Intake (mg/day, week)	PEDro Score
	M/F	N	Age (Years)			
Tucker et al. [28]	M	5	22 ± 1	professional	<500	8
Abian-Vicen et al. [29]	M	16	14.9 ± 0.8	professional	<60	9
Puente et al. [30]	M/F	10/10	27.1 ± 4.0/27.9 ± 6.1	professional	<100	9
Puente et al. [31]	M/F (AA/CC)	10/9	26.5 ± 2.4/27.0 ± 5.3	professional	<100	10
Scanlan et al. [32]	M/F	11/10	18.3 ± 3.3	professional	<100	10
Stojanović et al. [33]	F	10	20.2 ± 3.9	professional	<100	10
Tan et al. [34]	M/F	12/6	23.1 ± 1.9/22.0 ± 1.3	college	<200	10
Stojanović et al. [35]	M	11	16.5 ± 1.0	juniors	310 ± 76	10

M/F—male/female; N—number; AA—AA homozygotes; CC—allele carriers, PEDro—Physiotherapy Evidence Database Scale.

**Table 2:** Experimental program of acute caffeine ingestion in basketball players.

Study	Form of Caffeine	Dose (mg/kg)	Timing (min)	Variables (Unit)	Placebo	Caffeine	Results
Tucker et al. [28]	Capsule	3	60	VO2max (rep) 10VJ (cm)	118/126/109/122/83 ± 13/14/6/14/4	124/117/119/111/87 ± 5/13/9/9/4	↔ ↔
Abian-Vicen et al. [29]	Energy drink	3	60	FTA (%) TPA (%) CMJ (cm) 15—RJ (cm) YoYo IR 1 (m) UE (μg/mL)	70.7 ± 11.8 39.9 ± 11.8 37.5 ± 4.4 28.8 ± 3.4 1.925 ± 702 0.1± 0.1	70.3 ± 11.0 38.1 ± 12.8 38.3 ± 4.4 30.2 ± 3.6 2.000 ± 706 1.2 ± 0.7	↔ ↔ ↑ ↑ ↔ ↑
Puente et al. [30]	Capsule	3	60	VJ (cm) CODAT (s) CODATwb (s) FTA (%) FT (n) Reb (n) Ass (n) Imp (imp/min) PIR (%) POI (%)	37.3 ± 6.8 5.96 ± 0.29 6.20 ± 0.29 15.4 ± 1.6 0.6 ± 0.8 2.5 ± 2.0 1.1 ± 0.9 396 ± 43 7.2 ± 8.6 19.0	38.2 ± 7.4 5.95 ± 0.3 6.14 ± 0.32 15.6 ± 2.3 1.1 ± 1.1 3.7 ± 2.6 2.1 ± 1.6 410 ± 41 10.6 ± 7.1 54.4	↑ ↔ ↔ ↔ ↑ ↑ ↑ ↑ ↑ ↑
Puente et al. [31]	Capsule	3	60	VJ (cm) CODAT (s) CODATwb (s) Mhr (bpm) Phr (bpm) Imp (imp/min)	39.6 ± 7.2/36.3 ± 5.9 5.91 ± 0.25/5.95 ± 0.33 6.19 ± 0.21/6.14 ± 0.35 158 ± 9/161 ± 13 187 ± 12/182 ± 7 385 ± 48/401 ± 36	40.7 ± 7.3/37.2 ± 6.9 5.88 ± 0.27/5.97 ± 0.38 6.09 ± 0.24/5.97 ± 0.38 160 ± 10/163 ± 9 188 ± 13/185 ± 6 401 ± 36/415 ± 35	↑/↔ ↔ ↔ ↔ ↔ ↑/↑
Scanlan et al. [32]	Capsule	3	60	TDT20m (s) DD20m (s)	3.560 ± 0.184 0.145 ± 0.138	3.528 ± 0.208 0.150 ± 0.129	↔ ↔
Stojanović et al. [33]	Capsule	3	60	CMJ (cm) CMJa (cm) SJ (cm) LAT (s) 5 m sprint (s) 10 m sprint (s) 20 m sprint (s) 5 m sprint wb (s) 10 m sprint wb (s) 20 m sprint wb (s) RSA (s) RPE (AU) PP (AU)	27.92 ± 4.24 33.85 ± 3.92 25.97 ± 3.16 13.22 ± 0.87 1.24 ± 0.15 2.11 ± 0.18 3.59 ± 0.25 1.22 ± 0.08 2.07 ± 0.11 3.65 ± 0.15 32.20 ± 1.74 7.8 ± 1.2 3.6 ± 2.8	29.20 ± 4.39 35.14 ± 5.08 27.22 ± 4.37 12.99 ± 0.86 1.18 ± 0.11 2.01 ± 0.13 3.49 ± 0.23 1.20 ± 0.05 2.05 ± 0.12 3.56 ± 0.25 31.80 ± 1.62 5.6 ± 2.5 3.6 ± 2.8	↑ ↑ ↑ ↔ ↔ ↑ ↑ ↔ ↔ ↔ ↔ ↓ ↔
Tan et al. [34]	Capsule	6	60	FTA (%) HR (bpm) RPE (AU)	5.5 ± 2.0 163 ± 12.1 15.7 ± 2.1	6.1 ± 1.7 166 ± 9.2 15.8 ± 2.1	↔ ↑ ↔
Stojanović et al. [35]	Capsule	3	60	CMJ am/pm (cm) CMJa am/pm (cm) SJ am/pm (cm) LAT am/pm (s) 20 m sprint (s) RSA (s) RSAwb (s)	31.03 ± 4.98 (am) 39.98 ± 5.23 (am) 30.55 ± 4.89 (am) 12.61 ± 0.84 (pmc) X 29.91 ± 1.31 X	33.90 ± 5.38 (am) 42.32 ± 5.69 (am) 33.20 ± 4.71(am) 11.98 ± 0.70 (amc) X 26.49 ± 1.62 (amc) X	↑ ↑ ↑ ↑ ↑

VO2max—maximal oxygen uptake; VJ—vertical jumps; FTA—free throw accuracy; TPA—three point accuracy; CMJ—countermovement jump; RJ—repeated jumps; YoYo IR 1—yoyo intermittent recovery test; UE—urinary excretion; CODAT (wb)—change of direction agility test (with ball); Reb—rebounds; Ass—assists; Imp—body impacts; PIR—performance index; POI—prevalence of insomnia; Mhr—mean heart rate; Phr—peak heart rate; TDT—total dribbling time; DD—dribbling deficit; CMJa—countermovement jump with arm swing; SJ—squat jump; LAT—lane agility drill test; RSA (wb)—repeated sprint ability (with ball); RPE—perceived exertion; PP—performance; HR—heart rate; am—morning; pm—evening; amc—morning caffeine group; pmc—evening caffeine group; ↑—improvement; ↓—reduction; ↔—unchanged.

## Data Availability

No new data were created or analyzed in this study.
